# Supplementary figures and images for: Increased paternal corticosterone exposure influences offspring behaviour and expression of urinary pheromones
Source: BMC Biol. 2023 Sep 5;21:186. doi: 10.1186/s12915-023-01678-z (PMC10478242; doi:10.1186/s12915-023-01678-z)

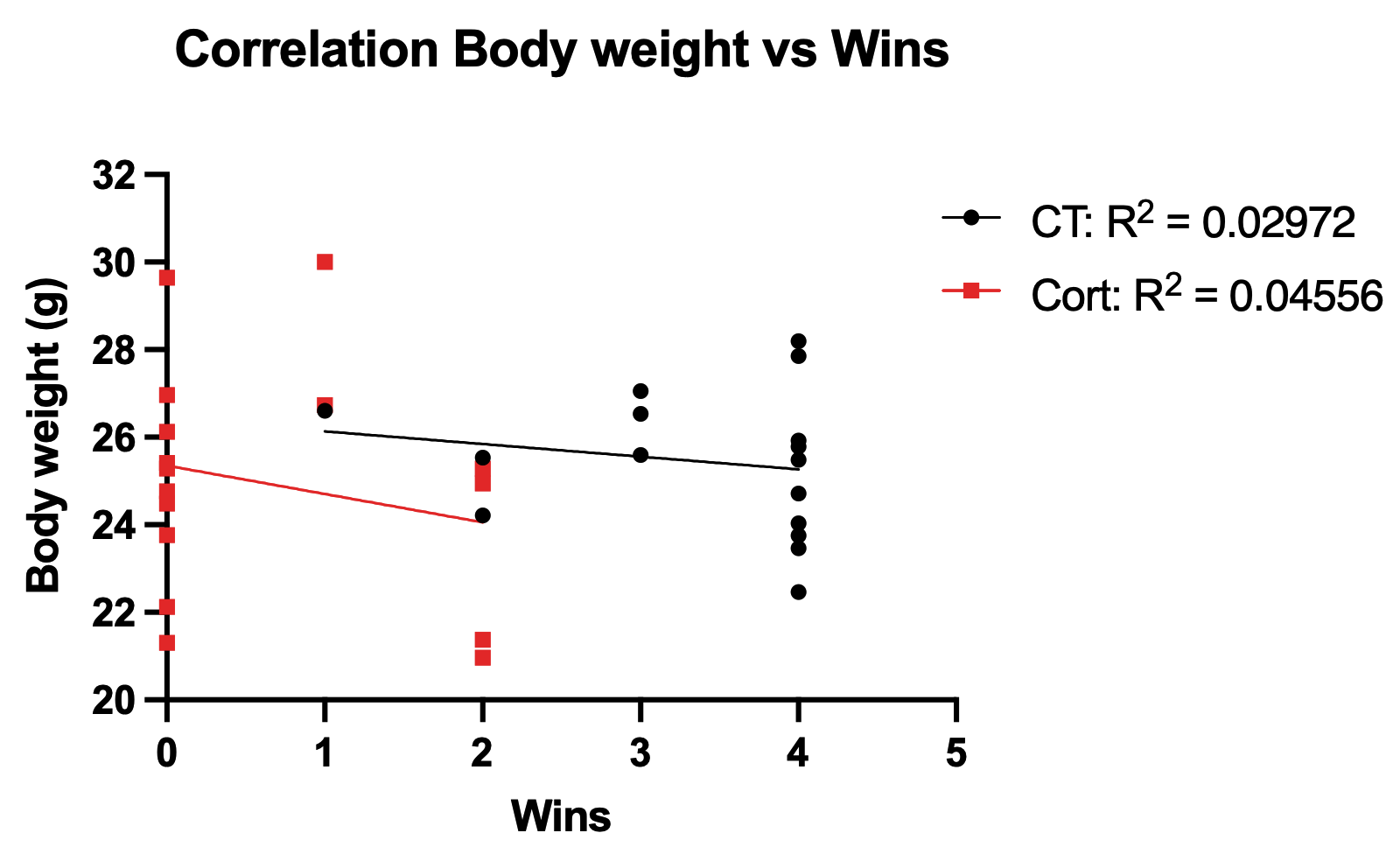

Supplement: Supplementary file 1 — Additional file 1: Fig. S1. Correlation between body weight and performance in the social dominance tube test. No statistically significant correlation was found for CT or Cort groups. Simple linear regression. CT p=0.5232, n=16; Cort p=0.4273, n=16. [file 12915_2023_1678_MOESM1_ESM.tiff]

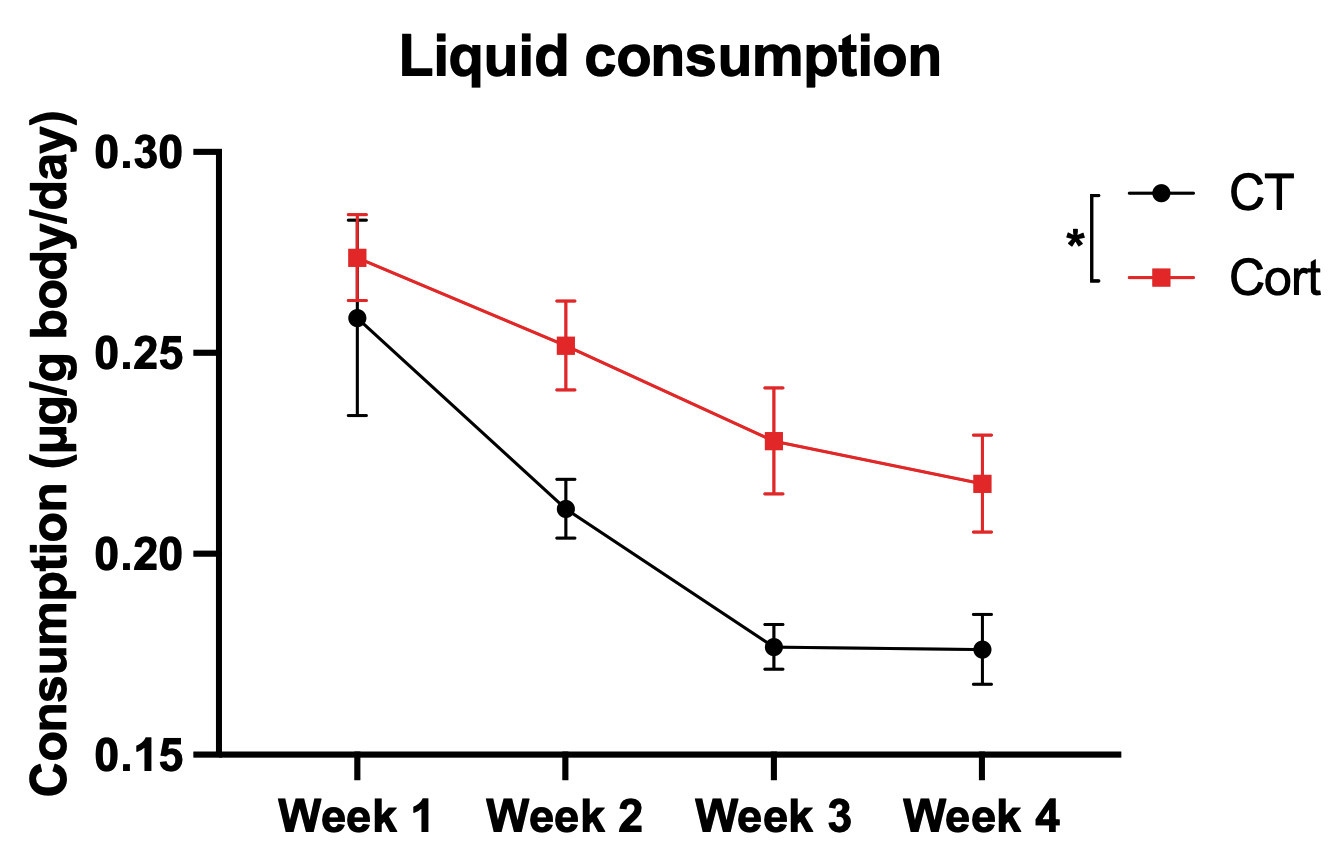

Supplement: Supplementary file 2 — Additional file 2: Fig. S2. Assessing liquid consumption during Corticosterone treatment. As expected, Cort-treated mice drink more liquid compared to CT. Repeated-measures ANOVA. CT n=16, Cort n=16. Group: F(1,30)=5.835, p=0.04388. * p<0.05. [file 12915_2023_1678_MOESM2_ESM.tiff]

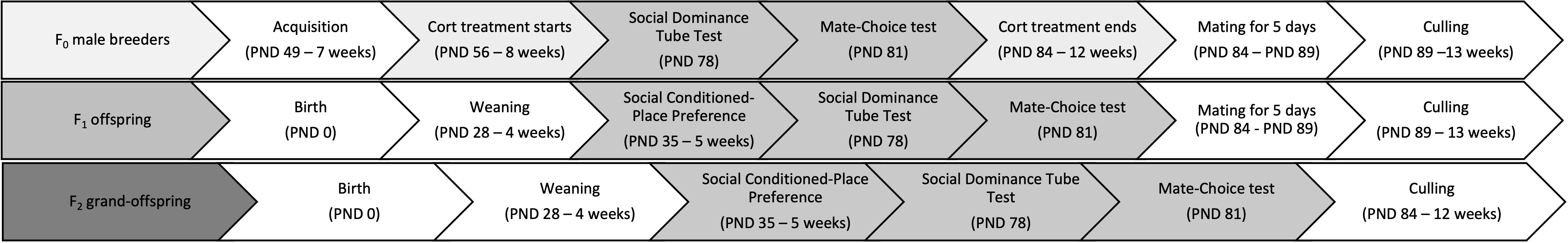

Supplement: Supplementary file 3 — Additional file 3: Fig. S3. Experimental design. [file 12915_2023_1678_MOESM3_ESM.jpg]
